# Association between baseline cardio-kidney-metabolic syndrome, its transition and cognitive impairment: result from CHARLS study

**DOI:** 10.1186/s13098-025-01779-5

**Published:** 2025-06-13

**Authors:** Yuanyue Zhu, Xuejie Wang, Kan Wang, Yiming Dai, Weiguo Hu, Yufang Bi, Linhui Shen

**Affiliations:** 1 Department of Geriatrics, Ruijin hospital, Shanghai Jiao Tong University School of Medicine, Shanghai, China; 2 Medical Center on Aging of Ruijin Hospital, Shanghai Jiao Tong University School of Medicine, Shanghai, China; 3 Department of Nephrology, Ruijin Hospital LuWan Branch, Shanghai Jiao Tong University School of Medicine, Shanghai, China; 4 Department of Endocrine and Metabolic Diseases, Shanghai Institute of Endocrine and Metabolic Diseases, Ruijin Hospital, Shanghai Jiao Tong University School of Medicine, Shanghai, China; 5 Shanghai National Clinical Research Center for Metabolic Diseases, Key Laboratory for Endocrine and Metabolic Diseases of the National Health Commission of the PR China, Shanghai Key Laboratory for Endocrine Tumor, Shanghai National Center for Translational Medicine, Ruijin Hospital, Shanghai Jiao Tong University School of Medicine, Shanghai, China; 6 Key Laboratory of Public Health Safety of Ministry of Education, Key Laboratory of Health Technology, School of Public Health, Assessment of National Health Commission, Fudan University, Shanghai, China

**Keywords:** Cardiovascular-Kidney-Metabolic syndrome, Transition, Cognitive impairment, Odds ratio

## Abstract

**Aims:**

Cardiovascular-Kidney-Metabolic (CKM) syndrome is an integrated context encompassing diabetes, obesity, cardiovascular, and chronic kidney diseases. The impact of CKM syndrome on cognitive impairment remained unclear.

**Methods:**

This longitudinal, observational study used data from the China Health and Retirement Longitudinal Study waves 1 and 4 (2011 to 2018). In total, 8,833 participants were included for the analysis between baseline CKM and cognitive impairment, and 4,230 were included for the analysis between CKM transition and cognitive impairment. Baseline CKM were classified into 5 consecutive stages according to the AHA statement, and transitions in CKM stages were classified as improved, stable, or progressed based on the difference in states between 2011 and 2015. Logistic regressions were used to explore the associations between CKM stages, transitions and the risk of subsequent cognitive impairment.

**Result:**

Compared with those in stage 0, the adjusted odds ratio [95% confidence intervals] (aOR [95% CI]) of incident cognitive impairment were 1.74 (1.00-3.18) for stage 1, 2.05 (1.17-3.81) for stage 2, 2.09 (1.27-3.66) for stage 3, and 3.91 (2.33-6.99) for stage 4, respectively. The odds ratios were higher among male and elder participants. In the transition analysis, the aOR (95% CI) was 0.44 (0.19-1.03) for improved group and 1.61 (1.01-2.59) for progressed group, compared with the those maintaining stable CKM stages.

**Conclusions:**

Higher CKM stages are associated with incrementally elevated risk of cognitive impairment. Additionally, the progression of CKM stages corresponded with greater hazards of cognitive impairment, while stage reversion might be associated with reduced risk.

**Supplementary Information:**

The online version contains supplementary material available at 10.1186/s13098-025-01779-5.

## Introduction

Cardiovascular-Kidney-Metabolic (CKM) syndrome is a new context proposed by the American Heart Association (AHA) in 2023 which refers to the interconnections of diabetes, obesity, cardiovascular, and chronic kidney diseases [1]. According to the AHA statement, CKM syndrome encompasses 5 consecutive stages from 0 to 4, reflecting different severity of CKM risk factors and multimorbidity. The prevalence of stage 3 and stage 4 of CKM syndrome has reached 5.4% and 9.2%, respectively in the USA during 2011-2020, posing great burden on public health [2]. Advanced CKM stages are associated with higher risk of adverse health outcomes, including cardiovascular disease (CVD) and premature mortality [3–5]. However, little is known about the relationship between existing CKM syndrome and other health outcomes. Moreover, three possible transitions of CKM stages might occur: stable, progressed or reversed in natural circumstances [6]. Despite the existing evidence, health consequences related to these transitions remained to be explored [7]. Since distinctive transformations of CKM stages may indicate varying disease risks and underlying health conditions, understanding how the changing patterns of CKM syndrome affect future health risk might allow for personalized interventions towards high-risk populations.

Cognitive impairment is a growing public health concern, with over 150 million individuals projected to be affected by 2050 [8]. Therefore, exploring modifiable risk factors associated with cognitive diseases is crucial to reducing the burden on public health. It is implied that cardiovascular and metabolic dysregulations are linked to cognitive decline and increased risk of dementia [9] [10]. Nevertheless, how the integrated CKM syndrome affects the cognitive function remained undiscovered, and transitions in the syndrome may represent another dimension of risk factors [11].

Therefore, the current investigation aims to assess the relationship between the baseline CKM syndrome and its transition with the risk of cognitive impairment, using data from a nationwide, prospective study in China.

## Materials and methods

### Study subjects

Our research utilized data from the China Health and Retirement Longitudinal Study (CHARLS) including the baseline survey (2011) and three follow-up waves (2013, 2015, and 2018). Briefly, it is a longitudinal cohort covering 450 communities across 28 provinces in China using a multistage, stratified random sampling strategy [12] [13]. 17,708 individuals were enrolled at baseline survey in 2011. In-person interviews and physical examinations were conducted at each wave, and biomedical measurements were completed at 2011 and 2015. This study has been approved by the Biomedical Ethics Committee of Peking University (No. IRB00001052-11015). All participants have provided written informed consent. This study was conducted according to the principles of the Helsinki Declaration.

The distributions of CKM stages at 2011 and 2015 were analyzed among those with complete data of CKM components. Therefore, participants with missing data for any of the variables required to define CKM were excluded from the analysis at both waves [2]. The assessment of cognitive impairment continued from baseline through 2018. For the analysis of baseline CKM stages and incident cognitive impairment, participants who had cognitive impairment at baseline (2011) or had missing information on cognitive impairment at wave 2–4 were excluded. For the analysis of CKM stage transitions, participants who had cognitive impairment at 2015 or missing information on cognitive impairment at wave 3–4 were excluded.

### Data collection

Information on medical history, demographic and lifestyle factors were obtained through validated questionnaires. Anthropometric measurements (systolic blood pressure (SBP), diastolic blood pressure (DBP), height, weight and waist circumference (WC)) were conducted at site by trained nurses. Laboratory tests (creatinine, total cholesterol (TC), high-density cholesterol (HDL-c), triglyceride (TG), hemoglobin A1c (HbA1c), fasting blood glucose (FBG)) were completed at local centers.

### Definitions of CKM components

SBP and DBP were recorded as the mean values of the three blood pressure measurements at each wave. Body mass index (BMI) was calculated as weight (kg) divided by height squared (m^2^). Obesity was defined as BMI ≥ 23 kg/m^2^, abdominal obesity was defined as waist circumference ≥ 80 cm for male and 90 cm for female [14]. Prediabetes was defined as FBG of 5.6–6.1 mmol/L, or HbA1C of 5.6-6.4% and without previous diagnosis of diabetes. Diabetes was defined as a self-reported history of diabetes, or current use of hypoglycemic drug, or FBG ≥ 7.0 mmol/L, or HbA1C ≥ 6.5% [15]. Hypertension was defined as a self-reported history of hypertension, or current use of antihypertension drug, or SBP ≥ 130 mmHg, or DBP ≥ 80 mmHg [16]. Dyslipidemia was defined as a self-reported history of dyslipidemia, or current use of lipid-lowering drugs, or a measured TC ≥ 5.2 mmol/L or LDL-c ≥ 3.4 mmol/L or HDL-c < 1.0 mmol/L or TG ≥ 1.7 mmol/L [17]. Metabolic syndrome was diagnosed if 3 or more of the following components existed: (i) abdominal obesity; (ii) low HDL-c: HDL-c < 50 mg/dL in female or < 40 mg/dL in male; (iii) high TG: TG ≥ 150 mg/dL; (iv) High blood pressure (SBP ≥ 130 mmHg or DBP ≥ 80 mmHg or antihypertension treatment); (v) FBG ≥ 5.6 mmol/L [18]. Chronic kidney disease was staged according to estimated glomerular filtration rate (eGFR) calculated from measured creatinine. While KDIGO guidelines recommend using both eGFR and albuminuria for staging, only eGFR was used in this study as the CHARLS study did not include urine albuminuria measurements [4] [19]. CVD was defined as a composite outcome of heart disease and stroke, based on their answers to the question “have you ever been diagnosed with stroke by your doctor?” and “have you ever been diagnosed with heart attack by your doctor?”. Clinical CVD was ascertained if one of the answers to these questions was “Yes” and medical records were required upon positive answers [20].

### Stages and transitions of CKM syndrome

The stages of CKM syndrome were defined according to the advisory released by AHA [21]and the diagnosis method was consistent across 2011 and 2015. Based on the recommendations, CKM syndrome was categorized into 5 progressive stages according to the medical history of CVD, laboratory tests, physical examinations and 10-year CVD risk predicted by the PERVENT algorithms [22]. Specifically, stage 0 referred to normal weight without metabolic abnormalities, stage 1 referred to the presence of obesity, or central obesity, or prediabetes, stage 2 referred to an elevated TG (≥ 135 mg/dL), or hypertension, or diabetes, or metabolic syndrome, or moderate-to-high-risk CKD, stage 3 referred to very-high-risk CKD stages, or a high-predicted 10-year CVD risk (≥ 20%) and stage 4 referred to a self-reported heart disease or stroke. Detailed criteria for the staging system of CKM syndrome is listed in the ESM Table 1 with available data from CHARLS study. Transition in CKM syndrome was defined as the changes through stages between 2011 and 2015. Participants having same stages at 2011 and 2015 were defined as *stable*. Those with higher stages at 2015 than 2011 were defined as *progressed*, and those with lower stages in 2015 than 2011 were determined as *improved*. Notably, individuals classified as stage 4 at baseline were excluded from the *transition analysis*, as they would not experience any transition due to the established medical history of CVD. This exclusion criterion ensured that the following analysis focused solely on individuals who underwent transition during follow up.

**Table 1:** Distribution of CKM stages at 2011 and 2015

					Stage 0	Stage 1	Stage 2	Stage 3	Stage 4
2011									
	Total				911 (9.8%)	1771 (19.1%)	4355 (47.0%)	933 (10.1%)	1288 (13.9%)
	Sex								
			Male		533 (12.6%)	800 (18.9%)	1785 (42.1%)	590 (13.9%)	535 (12.6%)
			Female		378 (7.5%)	971 (19.4%)	2570 (51.2%)	343 (6.8%)	753 (15.0%)
	Age								
			< 65		740 (10.8%)	1510 (22.0%)	3600 (52.3%)	174 (2.5%)	854 (12.4%)
			≥ 65		171 (7.2%)	261 (11.0%)	755 (31.7%)	759 (31.9%)	434 (18.2%)
2015									
	Total				304 (5.6%)	950 (17.5%)	2601 (48.0%)	441 (8.1%)	1121 (20.7%)
	Sex								
			Male		188 (7.6%)	492 (20.0%)	1033 (42.0%)	294 (12.0%)	451 (18.3%)
			Female		116 (3.9%)	458 (15.5%)	1568 (53.0%)	147 (5.0%)	670 (22.6%)
	Age								
			< 65		258 (6.2%)	810 (19.4%)	2225 (53.2%)	111 (2.7%)	777 (18.6%)
			≥ 65		46 (3.7%)	140 (11.3%)	376 (30.4%)	330 (26.7%)	344 (27.8%)

### Definition of cognitive impairment

Cognitive impairment was assessed based on the answer to the question “Have you been diagnosed with memory-related disease (including Alzheimer’s disease, brain atrophy, or Parkinson’s disease) by your doctor?”. The diagnosis was verified based on the diagnosis date and medical treatment, consistent to prior studies [23] [24]. For baseline analysis, incident cognitive impairment was defined as the first occurrence of memory-related disease identified after wave 1 (2011 baseline survey). For the transition analysis, incident cognitive impairment was defined as the first occurrence of memory-related disease after wave 3 (2015 survey).

### Covariates

Covariates were selected based on guidelines on dementia prevention and influential factors of CKM stages [25] [26]. Information on smoking and drinking behaviors, marital status, education levels, income, healthcare accessibility, and living environment were obtained from the questionnaires. Physical activity was assessed by the Chinese-version of International Physical Activity Questionnaire. Ideal physical activity was defined as a total physical activity of more than 600 METs/min per week [27]. Depressive symptoms were assessed using simplified Center for Epidemiologic Studies Depression Scale (CESD), with 10 scores or higher to define depression [28]. In accordance with previous studies, a comprehensive social determinants of health (SDOH) score were calculated based on social support, income, health insurance, and education level [26]. Participants were divided into four groups based on SODH scores and higher SODH quartiles indicated more disadvantaged socioeconomic status (SES).

### Statistical analysis

Baseline characteristics according to the baseline CKM stages and CKM transitions were separately presented. Continuous variables were described as means (standard deviations), and categorical variables were presented in numbers (proportion). Comparisons between groups were conducted using ANOVA for continuous variables and the Chi-square test for categorical variables. Logistic regressions were used to examine the associations between baseline CKM stages, CKM transitions, and the risk of cognitive impairment. Model 1 was the crude model, model 2 was adjusted for age and sex, and model 3 was further adjusted for current drinking (yes/no), ideal physical activity (yes/no), married (yes/no), depression (yes/no) and SODH categories at baseline. Multiple imputation was used to impute all missing covariates. Subgroup analyses by age and sex were further conducted to examine potential differences in these associations across demographic characteristics. Multi-group comparisons were made in pairs to control for Type I error. All analyses were conducted using R (version 4.4.1), and a two-tailed *P* < 0.05 was considered as statistically significant.

## Results

### Baseline characteristics of included participants at 2011 and 2015

**Fig. 1:**
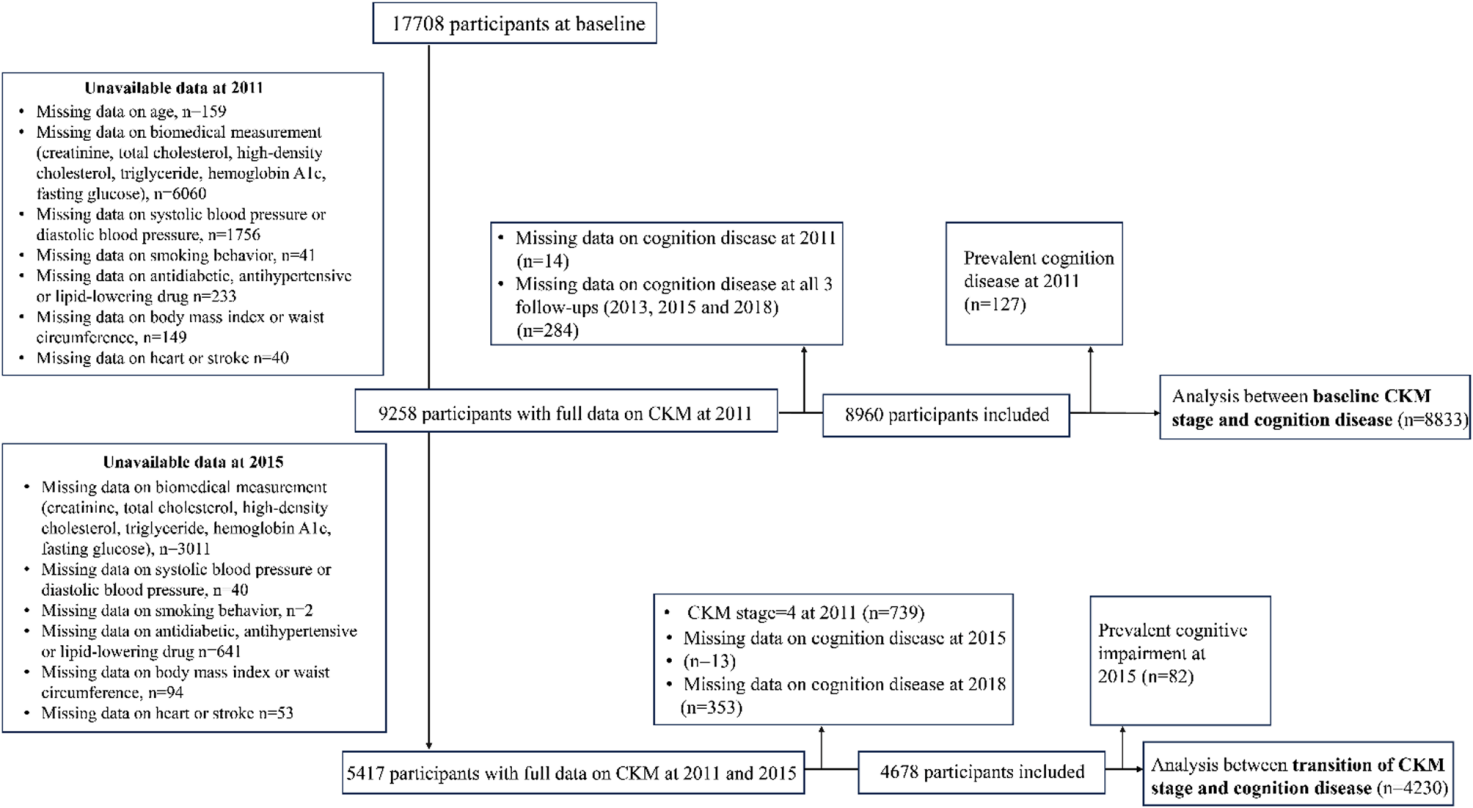
Flowchart of the study

The detailed exclusion process is shown in Fig. 1. In general, a total of 8,833 individuals (mean age 58.8 ± 9.3 years, 54.6% women) were included in the baseline CKM analysis and 4,866 (mean age 57.8 ± 8.6 years, 55.2% women) were included in the transition analysis. The overall incidence of cognitive impairment was 4.1%. Baseline characteristics are shown in ESM Tables 2 and ESM Table 3. Participants within advanced CKM stages at baseline were more likely to have disadvantaged SES, elder and exhibited lower levels of physical activity. Furthermore, these individuals typically displayed poorer metabolic health profiles and higher rates of depression. As to the transition analysis, those experiencing progression in CKM stages had higher percentages of current smokers and drinkers compared to those remaining *stable* or *improved*, and demonstrated greater prevalence of depression. Compared to participants included in the baseline analysis, those excluded from the analysis had worse lipid profiles and higher blood pressure, and less likely to be physical active or married (ESM Table 4).

**Table 2:** Baseline CKM stages and the risk of cognitive impairment

		Case (%)	Model 1	Model 2	Model 3	*p* for trend	
Total	Stage 0	16 (1.8)	ref (1.00)	ref (1.00)	ref (1.00)		
	Stage 1	48 (2.8)	1.57 (0.91-2.87)	1.74 (1.00-3.18)	1.74 (1.00-3.18)		
	Stage 2	146 (3.5)	1.97 (1.21-3.45)	2.05 (1.16-3.80)	2.05 (1.17-3.81)		
	Stage 3	63 (7.6)	4.51 (2.65-8.15)	2.07 (1.26-3.63)	2.09 (1.27-3.66)		
	Stage 4	92 (7.7)	4.57 (2.75-8.12)	3.80 (2.27-6.78)	3.91 (2.33-6.99)		< 0.001
Female	Stage 0	6 (3.2)	ref (1.00)	ref (1.00)	ref (1.00)		
	Stage 1	26 (13.8)	0.86 (0.41-1.91)	0.89 (0.43-1.99)	0.89 (0.43-1.99)		
	Stage 2	67 (35.6)	1.18 (0.64-2.45)	1.01 (0.45-2.38)	1.05 (0.47-2.48)		
	Stage 3	41 (21.8)	2.40 (1.24-5.09)	1.07 (0.57-2.22)	1.09 (0.58-2.27)		
	Stage 4	48 (25.5)	2.79 (1.33-6.24)	1.80 (0.92-3.86)	1.90 (0.97-4.09)		0.075
Male	Stage 0	10 (5.6)	ref (1.00)	ref (1.00)	ref (1.00)		
	Stage 1	22 (12.4)	2.99 (1.31-8.08)	3.17 (1.38-8.58)	3.19 (1.39-8.63)		
	Stage 2	79 (44.6)	3.50 (1.64-9.08)	3.73 (1.74-9.69)	3.75 (1.75-9.75)		
	Stage 3	22 (12.4)	7.35 (3.33-19.42)	3.90 (1.69-10.62)	3.81 (1.65-10.38)		
	Stage 4	44 (24.9)	9.38 (4.30-24.67)	7.58 (3.45-20.01)	7.62 (3.46-20.13)		< 0.001
< 65	Stage 0	13 (6.5)	ref (1.00)	ref (1.00)	ref (1.00)		
	Stage 1	30 (15)	1.14 (0.61-2.28)	1.20 (0.64-2.40)	1.22 (0.65-2.45)		
	Stage 2	97 (48.5)	1.57 (0.91-2.96)	1.66 (0.53-4.47)	1.64 (0.52-4.42)		
	Stage 3	5 (2.5)	1.72 (0.55-4.63)	1.67 (0.96-3.15)	1.70 (0.98-3.20)		
	Stage 4	55 (27.5)	4.01 (2.24-7.72)	4.35 (2.42-8.39)	4.29 (2.39-8.29)		0.035
> 65	Stage 0	3 (1.8)	ref (1.00)	ref (1.00)	ref (1.00)		
	Stage 1	18 (10.9)	3.94 (1.42-16.36)	3.98 (1.43-16.57)	4.01 (1.44-16.68)		
	Stage 2	49 (29.7)	4.27 (1.42-18.46)	4.29 (1.42-18.55)	4.37 (1.44-18.88)		
	Stage 3	58 (35.2)	5.15 (1.87-21.31)	5.16 (1.88-21.35)	5.14 (1.86-21.29)		
	Stage 4	37 (22.4)	5.71 (2.02-23.88)	5.74 (2.03-24.05)	5.93 (2.09-24.91)		< 0.001

Model 1: crude model

Model 2: adjusted for age and sex

Model 3: further adjusted for current drinking (yes/no), ideal physical activity (yes/no), married (yes/no), depression (yes/no) and quartiles of social determinants of health

**Table 3:** CKM transitions and the risk of cognitive impairment

	Improved	Stable	Progressed
**Total population**			
Case (%)	6 (1.2)	61 (2.5)	33 (2.7)
Model 1	0.47 (0.20-1.11)	ref (1.00)	1.08 (0.71-1.66)
Model 2	0.49 (0.21-1.15)	ref (1.00)	1.12 (0.73-1.73)
Model 3	0.51 (0.21-1.16)	ref (1.00)	1.13 (0.73-1.74)
Model 4	0.44 (0.19-1.03)	ref (1.00)	1.61 (1.01-2.59)
**Stage 0–1**			
Case (%)	1 (1.1%)	11 (2.0%)	10 (1.1%)
Model 1	0.56 (0.03-2.93)	ref (1.00)	0.56 (0.23-1.33)
Model 2	0.59 (0.03-3.09)	ref (1.00)	0.55 (0.23-1.32)
Model 3	0.57 (0.03-3.02)	ref (1.00)	0.56 (0.23-1.33)
**Stage 2**			
Case (%)	3 (1.0%)	36 (2.1%)	15 (4.7%)
Model 1	0.45 (0.11-1.26)	ref (1.00)	2.30 (1.21-4.17)
Model 2	0.45 (0.11-1.27)	ref (1.00)	1.67 (0.86-3.12)
Model 3	0.45 (0.11-1.28)	ref (1.00)	1.71 (0.87-3.19)
**Stage 3**			
Case (%)	2 (1.9%)	14 (6.5%)	8 (20.0%)
Model 1	0.28 (0.04-1.02)	ref (1.00)	3.62 (1.35-9.19)
Model 2	0.41 (0.06-1.69)	ref (1.00)	4.25 (1.52-11.45)
Model 3	0.41 (0.06-1.68)	ref (1.00)	4.62 (1.63-12.75)

Model 1: crude model

Model 2: adjusted for age and sex

Model 3: further adjusted for current drinking (yes/no), ideal physical activity (yes/no), married (yes/no), depression (yes/no) and quartiles of social determinants of health

### Distribution of CKM stages and transitions

Prevalence of CKM stages by age and sex in 2011 and 2015 are presented in Table 1. In 2011, stage 0 to stage 4 accounted for 9.8%, 19.1%, 47.0%, 10.1% and 13.9% of all enrolled participants, respectively. The distribution slightly shifted in 2015, with the proportions of stage 0 to 4 being 5.6%, 17.5%, 48.0%, 8.1% and 20.7%. Sex-specific description revealed that female participants had higher proportions of stage 4 compared to male counterparts. Additionally, the proportion of stage 3 and stage 4 were significantly higher among the elderly compared with younger individuals.

**Fig. 2:**
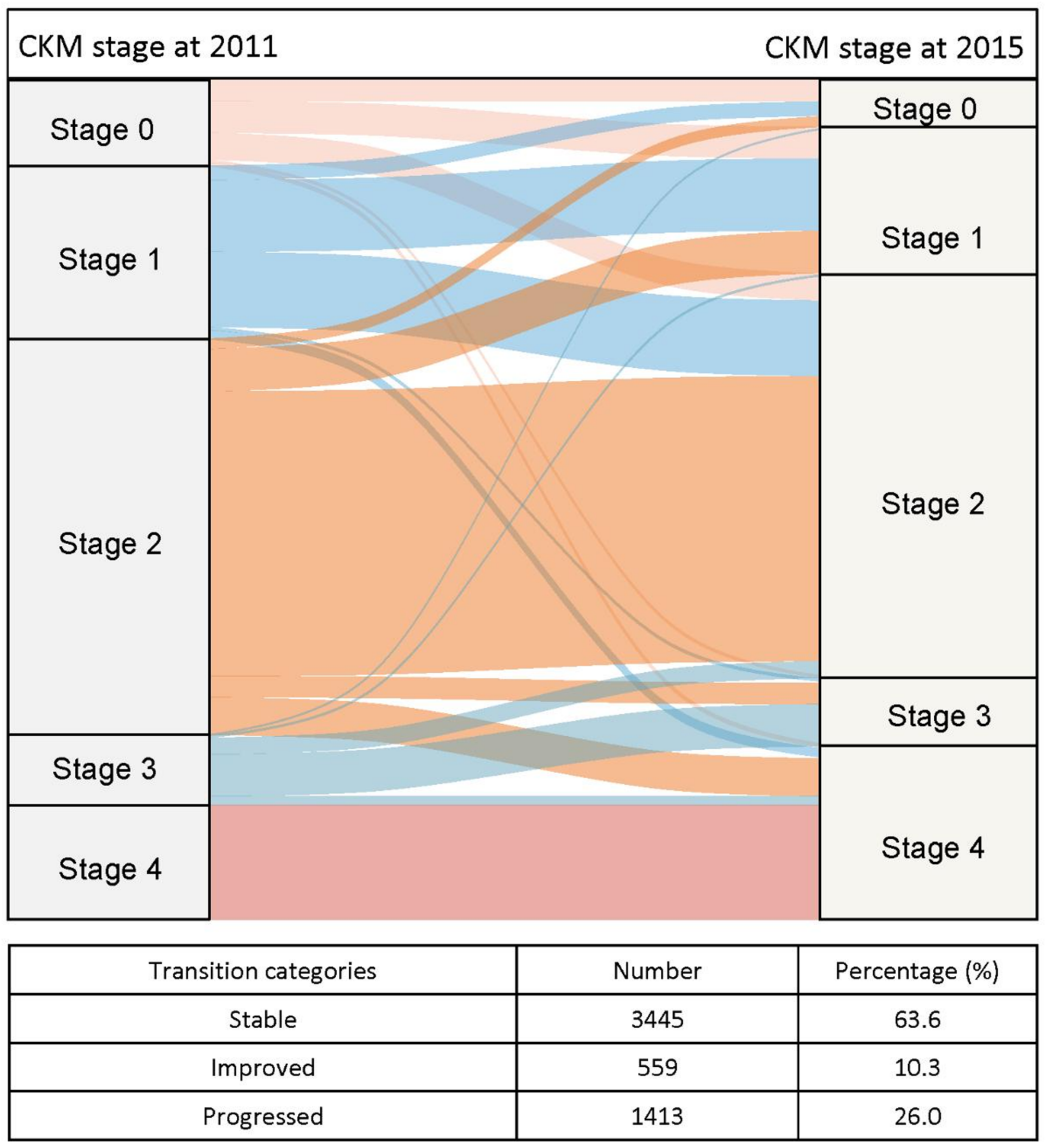
Transitions of CKM stages

The global transition patterns of CKM stages from 2011 to 2015 are visualized in the Sankey diagram (Fig. 2). The majority of participants remained stable in CKM stage, accounting for 63.6% for all the respondents in 2015. Additionally, 1413 individuals (26.1%) experienced a worsening in CKM stages, and 559 individuals (10.3%) achieved improvement.

### Association between baseline CKM stages and cognitive impairment

With CKM stages elevated, the risk of incident cognitive impairment also increased accordingly (Table 2). In the fully adjusted model, the adjusted odds ratio [95% confidence intervals] (aOR [95% CI]) of incident cognitive impairment were 1.74 [1.00-3.18] for stage 1, 2.05 [1.17-3.81] for stage 2, 2.09 [1.27-3.66] for stage 3, and 3.91 [2.33-6.99] for stage 4 compared with individuals in stage 0.

Subgroup analysis revealed heterogeneous effects by age and gender. For male individuals, the aOR [95% CI] of incident cognitive impairment for stage 1–4 were 3.19 [1.39-8.63], 3.75 [1.75-9.75], 3.81[1.65-10.38] and 7.62 [3.46-20.13]. However, cognitive risks were not significantly increased among female participants in the adjusted models. The participants aged over 65 years presented higher risk than younger counterparts, with the aOR [95% CI] of 4.01 [1.44-16.68] for stage 1, 4.37 [1.44-18.88] for stage 2, 5.14 [1.86-21.29] for stage 3, and 5.93 [2.09-24.91] for stage 4. While in those age < 65 years old, only stage 4 was associated with a significant 3.29-fold increase in the risk of cognitive decline, while stage 1–3 showed no significant correlation with cognitive impairment. Multiple comparisons analysis reinforced the robustness of the associations found in the primary analysis (ESM Table 5).

### Association between transition in CKM stage and cognitive impairment

Since the majority of participants remained in the same CKM category in 2015 as with 2011, the *stable* group was used as the reference to explore the effect of transition in CKM stages on the risk of cognitive impairment. Table 3 shows the relationship of CKM transitions with incident cognitive impairment in the general population and subgroups. Compared with those maintaining in the same stage, the aOR [95% CI] was 0.44 [0.19-1.03] for *improved* group. In contrast, those who experienced progression in CKM stages had a higher risk (aOR: 1.61, 95% CI:1.01-2.59). Varying effects of the transitions were discovered through a subgroup analysis based on baseline CKM stages. For individuals within stage 0–1 at baseline, no statistically significant difference was found in the odds ratios of cognitive impairment among “*improved*” and “*progressed*” group, with the aOR [95% CI] of 0.57 [0.30-3.02] and 0.56 [0.23-1.33], respectively. However, for those in relatively advanced stages [i.e., stage 2 and stage 3], consistently elevated risks were observed for the “*progressed*” group. Specifically, the aORs [95% CIs] were 1.71 [0.87-3.19] for individuals at baseline stage 2 and 4.62 [1.63-12.75] for those at baseline stage 3. In-pair comparison verified the detrimental effects of CKM progression and possible protective effects of improvement (ESM Table 6). When combing the *stable* and *improved* group together as the reference, progressed group had significantly higher risk of cognitive impairment, except for those within stage 0–1 at baseline (ESM Table 7).

## Discussion

Our study illustrates that the presence of CKM syndrome increases the risk of cognitive impairment in a dose-dependent manner. Importantly, the transition analysis reveal that the risk was significantly increased in those progressing to more advanced CKM stages, and improvement in CKM stages might be associated with lower risk of cognitive impairment.

The Sankey plot demonstrated the natural transitions of CKM among middle-aged and elderly populations. The majority of participants remained in the stable group, followed by those who progressed, while the fewest participants experienced a reversal. This observation was consistent with previous studies [6] [7]highlighting the difficulty of reverting to a more normal CKM state especially when no specific intervention is implemented. This progressive nature underscores the importance of elucidating the health consequences of poor CKM status.

Previous researches have described the association between individual components of CKM syndrome, cardiovascular multimorbidities and dementia, while the results are not entirely consistent [29]. Despite the well-recognized association between cardiovascular diseases and cognitive impairment, the neurological effects of other components of CKM are controversial [30] [31]. For example, although obesity has been reported to increase the risk of dementia by 30–60% [32, 33], some recent studies determined that obesity might be protective factors of cognition, e.g., “obesity paradox” [34]. Possible explanation could be reverse causation bias, where preclinical disease causes weight loss before formal dementia diagnosis [35]. Furthermore, the association between kidney function and cognition is more ambiguous [36] [37]. As a combination of all these cardio-renal-metabolic factors, the relationship between CKM and cognition still remains unclear [38] [39]. With data from a nationwide aging cohort, our study offers a more comprehensive assessment of CKM severity and cognition. Consistent with a study conducted in the UK Biobank, we found that the risk of cognitive impairment increases proportionally with the number of adverse renal-cardiometabolic factors [40]. According to the subgroup analysis, the associations between CKM stages and cognitive impairment were stronger among male and the elderly. While in several previous study conducted in European ancestries, younger and female participants were more vulnerable to cardiometabolic risk factors concerning dementia risk [41] [42]. These inconsistent results may be attributed to demographic and ethnicity differences, indicating a need for more specific prevention strategies for Chinese adults.

Furthermore, our analysis demonstrated that adverse transitions in CKM stages were associated with elevated risk of cognitive impairment. Although Frentz et al. reported that the change in metabolic status did not correspond with increased dementia risk [43], emerging evidence has established declining cardiometabolic factors as significant predictors of cognitive impairment [44] [45]. Importantly, our data also revealed that reversion from advanced to more favorable CKM stages might mitigate the established cognitive risk [46]. These findings correspond to previous reports that the risk of dementia was reduced when cardiometabolic risk factors were well managed [47] [48]. It is indicated that strategies to improve renal-cardiometabolic status may help to prevent cognitive impairment [49].

Mechanistically, the pathological processes underlying CKM components operate through multiple interconnected pathways that collectively contribute to cognitive impairment [9]. Specifically, insulin resistance is a fundamental feature relating CKM with cognitive impairment through increased amyloid-beta accumulation and elevated inflammation status [50]. Moreover, hypertension, dyslipidemia and other cardiovascular risk factors collectively cause small vessel dysfunction and reduced cerebral perfusion, leading to white matter damage and cognitive deficits [51]. More recently, dysregulated gut microbiota, and neuroinflammation have been proposed as potential mechanisms as well [52]. Contrarily, metabolic fitness could improve cognitive performance through acetate pathways [53]. However, future studies are warranted to verify these findings despite these speculations.

The present investigation is the first longitudinal study exploring baseline CKM stages, transitions in CKM and the risk of cognitive impairment. Nonetheless, it also has several limitations to be addressed. Firstly, selection bias represents a significant concern, as differences in several variables between included and excluded participants were observed. Secondly, the lack of UACR data may lead to underestimation of early-stage CKD prevalence although previous research has supported the utility of eGFR-only approaches for population-level CKD staging [4] [54]. Thirdly, since CHARLS dataset does not collect data on APOE status, we are unable to explain the results from genetic perspective. Fourthly, the reliance on self-reported cognitive impairment rather than standardized cognitive testing represents a significant limitation, despite validation through medical records and treatment information [23] [55]. Last but not least, the relatively short follow-up period may not be sufficient to detect meaningful long-term changes in cognitive impairment. This constraint should be considered when interpreting the findings, as cognitive impairment typically develops over decades.

In conclusion, the present study underscores the importance of addressing advanced CKM stages at baseline and monitoring disease progression, as both factors are associated with an increased risk of cognitive impairment.

## Electronic supplementary material

Below is the link to the electronic supplementary material.

Supplementary Material 1

## Data Availability

The original data was retrieved from Charls study (website: https://charls.pku.edu.cn/). Dataset of this analysis is available upon reasonable request.
