# Supplementary material for: Association between baseline cardio-kidney-metabolic syndrome, its transition and cognitive impairment: result from CHARLS study
Source: Diabetol Metab Syndr. 2025 Jun 13;17:211. doi: 10.1186/s13098-025-01779-5 (PMC12164072; doi:10.1186/s13098-025-01779-5)
Supplement: Supplementary file 1 — Supplementary Material 1 [file 13098_2025_1779_MOESM1_ESM.docx]

| **Supplementary files** |
| --- |
| ESM Table 1. CKM definition in CHARLS |
| ESM Table 2. Baseline characteristics according to CKM stages at 2011 |
| ESM Table 3. Baseline characteristics according to CKM transitions between 2011 and 2015 |
| ESM Table 4. Baseline characteristics of included and excluded participants |
| ESM Table 5. The Association between CKM stages and cognitive impairment with multiple comparisons |
| ESM Table 6. The Association between CKM transitions and cognitive impairment with multiple comparisons |
| ESM Table 7. The Association between combined CKM transitions and cognitive impairment |
|  |

| **ESM Table 1. CKM definition in CHARLS** | | |
| --- | --- | --- |
| CKM stages | Criterion | Threshold for CKM conditions |
| Stage 0 | All criteria are met | BMI <23 kg/m2 |
|  |  | Waist circumference <80/90 cm in female/male |
|  |  | Fasting blood glucose < 100 mg/dL and without self-reported diabetes or use of insulin/ oral hypoglycemic agents |
|  |  | SBP <130 mm Hg and DBP <80 mm Hg without self-reported |
|  |  | Diagnosis of hypertension/ use of antihypertensive medications |
|  |  | HDL cholesterol <50/40 mg/dL in female/male and triglycerides <150 mg/dL |
|  |  | Low-risk CKD in KDIGO classification: eGFR ≥ 60 ml/min/1.73m2 |
|  |  | Predicted 10-year CVD risk < 20% |
|  |  | No clinical CVD |
| Stage 1 | Any of the three criteria is met | Overweight/obesity: BMI ≥23 kg/m2 |
|  |  | Abdominal obesity: waist circumference <80/90 cm in female/male |
|  |  | Prediabetes |
|  | All criteria are met | SBP <130 mm Hg and DBP <80 mm Hg without self-reported |
|  |  | Diagnosis of hypertension or use of antihypertensive medications |
|  |  | HDL cholesterol <50/40 mg/dL in female/male and triglycerides <150 mg/dL |
|  |  | Low-risk CKD in KDIGO classification according to eGFR and |
|  |  | UACR: UACR < 30 mg/g and eGFR ≥ 60 ml/min/1.73m2. |
|  |  | Predicted 10-year CVD risk < 20% |
|  |  | No clinical CVD |
| Stage 2 | Any of the five criteria is met | Hypertriglyceridemia |
|  |  | Hypertension |
|  |  | Diabetes |
|  |  | Metabolic syndrome |
|  |  | Moderate-to-high-risk CKD in KDIGO classification |
|  | All criteria are met | No very high-risk CKD in KDIGO classification |
|  |  | Predicted 10-year CVD risk < 20% |
|  |  | No clinical CVD |
| Stage 3 | Any of the two criteria is met | Very high-risk CKD in KDIGO classification |
|  |  | Predicted 10-year CVD risk ≥ 20% |
|  | Any of the eight criteria is met | Overweight/obesity |
|  |  | Abdominal obesity |
|  |  | Prediabetes |
|  |  | Hypertriglyceridemia |
|  |  | Hypertension |
|  |  | Diabetes |
|  |  | Metabolic syndrome |
|  |  | Moderate-to-high-risk CKD in KDIGO classification |
|  | The criterion is met | No clinical CVD |
| Stage 4 | The criterion is met | Clinical CVD |

| **ESM Table 2. Baseline characteristics according to CKM stages at 2011** | | | | | | | |
| --- | --- | --- | --- | --- | --- | --- | --- |
|  | **Total** | **Stage 0** | **Stage 1** | **Stage 2** | **Stage 3** | **Stage 4** | **p value** |
| Number | 8833 | 892 | 1718 | 4202 | 827 | 1194 |  |
| SODH category |  |  |  |  |  |  |  |
| 1 | 2053 (23.2) | 205 (22.5) | 405 (22.9) | 1021 (23.4) | 183 (19.6) | 328 (25.5) | 0.019 |
| 2 | 2226 (25.2) | 250 (27.4) | 453 (25.6) | 1084 (24.9) | 231 (24.8) | 320 (24.8) |  |
| 3 | 2289 (25.9) | 240 (26.3) | 458 (25.9) | 1131 (26.0) | 245 (26.3) | 328 (25.5) |  |
| 4 | 2265 (25.6) | 216 (23.7) | 455 (25.7) | 1119 (25.7) | 274 (29.4) | 312 (24.2) |  |
| Female sex (%) | 4822 (54.6) | 378 (41.5) | 971 (54.8) | 2570 (59.0) | 343 (36.8) | 753 (58.5) | <0.001 |
| Current smoking (%) | 6204 (70.2) | 390 (42.8) | 519 (29.3) | 1109 (25.5) | 444 (47.6) | 313 (24.3) | <0.001 |
| Current drinking (%) | 5958 (67.5) | 371 (40.7) | 605 (34.2) | 1399 (32.1) | 313 (33.5) | 316 (24.5) | <0.001 |
| Ideal physical activity (%) | 1227 (32.5) | 291 (75.6) | 601 (76.5) | 1272 (67.9) | 185 (50.5) | 293 (53.3) | <0.001 |
| Married (%) | 1021 (11.6) | 833 (91.4) | 1625 (91.8) | 3931 (90.3) | 664 (71.2) | 1097 (85.2) | <0.001 |
| Antihypertensive drug (%) | 7171 (81.2) | 0 (0.0) | 0 (0.0) | 909 (20.9) | 357 (38.3) | 542 (42.1) | <0.001 |
| Antidiabetic drug (%) | 8493 (96.2) | 0 (0.0) | 0 (0.0) | 176 (4.0) | 74 (7.9) | 120 (9.3) | <0.001 |
| Lipid-lowering drug (%) | 8387 (95.0) | 3 (0.3) | 28 (1.6) | 204 (4.7) | 56 (6.0) | 195 (15.1) | <0.001 |
| Incident cognition impairment (%) | 365 (4.1) | 16 (1.8) | 48 (2.8) | 146 (3.5) | 63 (7.6) | 92 (7.7) | <0.001 |
| Age (yrs) | 58.8±9.3 | 56.9±8.5 | 55.9±8.1 | 57.3±7.9 | 72.8±8.2 | 61.5±9.2 | <0.001 |
| BMI (kg/m²) | 24.0±28.0 | 20.19±1.9 | 23.0±3.4 | 23.3±39.8 | 22.8±4.2 | 24.4±4.5 | <0.001 |
| Waist circumference (cm) | 84.3±12.4 | 74.6±9.0 | 82.0±10.9 | 86.3±12.2 | 85.4±12.6 | 86.7±13.8 | <0.001 |
| FBG (mmol/L) | 110.0±36.1 | 90.3±8.1 | 100.5±11.3 | 113.7±39.7 | 126.2±53.2 | 114.7±41.6 | <0.001 |
| HbA1C (%) | 5.2±0.8 | 5.0±0.4 | 5.1±0.4 | 5.3±0.9 | 5.5±1.1 | 5.4±0.9 | <0.001 |
| HDL (mg/dL) | 51.2±15.2 | 58.4±14.7 | 57.8±14.0 | 48.3±14.7 | 48.2±15.0 | 48.94±15.2 | <0.001 |
| TC (mg/dL) | 193.6±38.5 | 179.9±31.4 | 188.5±34.8 | 198.0±39.4 | 191.6±40.3 | 196.0±40.7 | <0.001 |
| TG (mg/dL) | 133.0±105.1 | 78.6±24.9 | 82.8±24.8 | 155.3±104.5 | 155.3±169.3 | 146.3±106.1 | <0.001 |
| SBP (mmHg) | 131.3±30.4 | 115.0±11.7 | 116.6±10.8 | 135.4±32.0 | 153.6±46.9 | 135.5±28.5 | <0.001 |
| eGFR (ml/min/1.73m²) | 92.4±14.8 | 96.4±11.8 | 96.6±12.0 | 93.5±13.8 | 78.5±17.5 | 87.9±16.2 | <0.001 |
| MMSE scores | 14.8±4.6 | 15.1±4.2 | 15.4±4.6 | 15.0±4.6 | 12.56±4.8 | 14.5±4.5 | <0.001 |
| CESD scores | 8.4±6.3 | 8.4±6.2 | 8.1±6.1 | 8.01±6.1 | 8.45±6.2 | 10.4±6.8 | <0.001 |

Continuous variables are presented in mean± standard deviations, categorical variables are presented in case (%).

SBP, systolic blood pressure; DBP, diastolic blood pressure; WC, waist circumference; TC, total cholesterol; HDL-c, high-density cholesterol; TG, triglyceride; HbA1c, hemoglobin A1c; FBG, fasting blood glucose.

| **ESM Table 3. Baseline characteristics according to CKM transitions between 2011 and 2015** | | | | | |
| --- | --- | --- | --- | --- | --- |
|  | **Total** | **Improved** | **Stable** | **Progressed** | **p value** |
| Number | 4230 | 507 | 2478 | 1245 |  |
| SODH category |  |  |  |  |  |
| 1 | 953 (22.5) | 561 (22.6) | 107 (21.1) | 285 (22.9) | 0.022 |
| 2 | 1072 (25.3) | 634 (25.6) | 134 (26.4) | 304 (24.4) |  |
| 3 | 1136 (26.9) | 653 (26.4) | 137 (27.0) | 346 (27.8) |  |
| 4 | 1069 (25.3) | 630 (25.4) | 129 (25.4) | 310 (24.9) |  |
| Female sex (%) | 2335 (55.2) | 1432 (57.8) | 225 (44.4) | 678 (54.5) | <0.001 |
| Current smoking (%) | 3084 (72.9) | 1872 (75.5) | 328 (64.7) | 884 (71.0) | <0.001 |
| Current drinking (%) | 2778 (65.7) | 1650 (66.6) | 314 (61.9) | 884 (71.0) | 0.129 |
| Ideal physical activity (%) | 630 (30.0) | 394 (31.5) | 71 (28.4) | 165 (27.5) | 0.171 |
| Married (%) | 400 (9.5) | 226 (9.1) | 53 (10.5) | 121 (9.7) | 0.602 |
| Antihypertensive drug (%) | 3276 (77.4) | 1812 (73.1) | 457 (90.1) | 1007 (80.9) | <0.001 |
| Antidiabetic drug (%) | 4009 (94.8) | 2322 (93.7) | 496 (97.8) | 1191 (95.7) | <0.001 |
| Lipid-lowering drug (%) | 3954 (93.5) | 2311 (93.0) | 484 (95.5) | 1159 (93.1) | <0.001 |
| Incident cognition impairment (%) | 200 (2.4) | 6 (1.2) | 61 (2.5) | 33 (2.7) | 0.0165 |
| Age (yrs) | 57.8±8.6 | 57.8±8.8 | 57.7±8.4 | 57.7±8.4 | 0.896 |
| BMI (kg/m²) | 24.3±16.5 | 24.5±14.9 | 22.6±3.6 | 24.5±21.8 | 0.052 |
| Waist circumference (cm) | 84.7±13.3 | 85.9±13.2 | 80.8±12.8 | 83.7±13.1 | <0.001 |
| FBG (mmol/L) | 102.9±32.9 | 105.3±36.4 | 95.7±26.6 | 100.9±26.9 | <0.001 |
| HbA1C (%) | 5.9±0.9 | 6.0±1.1 | 5.7±0.8 | 5.9±0.7 | <0.001 |
| HDL (mg/dL) | 51.7±12.1 | 51.2±12.0 | 52.9±11.3 | 52.1±12.8 | <0.001 |
| TC (mg/dL) | 184.9±36.6 | 187.71±37.56 | 178.3±35.2 | 182.2±34.72 | <0.001 |
| TG (mg/dL) | 142.1±90.6 | 153.33±97.28 | 106.9±69.9 | 134.1±79.14 | <0.001 |
| SBP (mmHg) | 129.5±30.4 | 131.66±32.50 | 122.2±15.9 | 128.3±30.1 | <0.001 |
| eGFR (ml/min/1.73m²) | 91.7±16.2 | 92.26±15.55 | 93.2±12.7 | 90.2±18.5 | <0.001 |
| MMSE scores | 14.5±4.8 | 14.53±4.90 | 14.4±4.5 | 14.6±4.7 | 0.836 |
| CESD scores | 7.9±6.2 | 7.76±6.11 | 7.5±6.1 | 8.5±6.4 | 0.001 |

Continuous variables are presented in mean± standard deviations, categorical variables are presented in case (%).

SBP, systolic blood pressure; DBP, diastolic blood pressure; WC, waist circumference; TC, total cholesterol; HDL-c, high-density cholesterol; TG, triglyceride; HbA1c, hemoglobin A1c; FBG, fasting blood glucose.

| **ESM Table 4. Baseline characteristics of included and excluded participants** | | | |
| --- | --- | --- | --- |
|  | **Included** | **Excluded** | **P value** |
| SODH category | 9258 | 8875 |  |
| 1 | 2142 (23.1) | 2285 (27.0) | <0.001 |
| 2 | 2338 (25.3) | 2089 (24.7) |  |
| 3 | 2402 (25.9) | 2025 (24.0) |  |
| 4 | 2376 (25.7) | 2051 (24.3) |  |
| Sex (female) | 5015 (54.2) | 4213 (49.9) | 0.331 |
| Current smoking | 2775 (30.0) | 2096 (27.4) | 0.229 |
| Current drinking | 3004 (32.4) | 2763 (33.3) | 0.097 |
| Ideal physical activity | 2642 (66.7) | 1770 (60.5) | 0.001 |
| Married | 8150 (88.0) | 7267 (86.3) | <0.001 |
| Antihypertensive drug | 1808 (19.5) | 1606 (19.6) | 0.445 |
| Antidiabetic drug | 370 (4.0) | 342 (4.2) | 0.575 |
| Lipid-lowering drug | 486 (5.2) | 379 (4.8) | 0.163 |
| Incident cognition impairment (%) | 135 (1.5) | 198 (2.4) | <0.001 |
| Age | 59.1±9.5 | 58.8±10.7 | 0.053 |
| BMI (kg/m²) | 23.9±27.4 | 24.5±49.0 | 0.445 |
| Waist circumference (cm) | 84.3±12.5 | 84.2±12.8 | 0.581 |
| FBG (mmol/L) | 110.2±37.3 | 110.5±37.6 | 0.737 |
| HbA1C (%) | 5.3±0.8 | 5.3±0.9 | 0.270 |
| HDL (mg/dL) | 51.2±15.3 | 49.4±15.4 | 0.125 |
| TC (mg/dL) | 193.5±38.5 | 190.9±40.2 | 0.004 |
| TG (mg/dL) | 132.6±104.2 | 143.8±130.9 | 0.357 |
| SBP (mmHg) | 131.6±31.3 | 142.7±99.4 | <0.001 |
| eGFR (ml/min/1.73m²) | 92.1±15.0 | 92.1±15.8 | 0.977 |
| MMSE score | 12. 8±2.9 | 12.1±3.2 | 0.150 |
| CESD scores | 8.31±6.35 | 8.22±6.01 | 0.552 |

Continuous variables are presented in mean± standard deviations, categorical variables are presented in case (%).

SBP, systolic blood pressure; DBP, diastolic blood pressure; WC, waist circumference; TC, total cholesterol; HDL-c, high-density cholesterol; TG, triglyceride; HbA1c, hemoglobin A1c; FBG, fasting blood glucose.

| **ESM Table 5. The Association between CKM stages and cognitive impairment with multiple comparisons** | | | |
| --- | --- | --- | --- |
|  | Pairwise comparisons | Estimates | Bonferroni-corrected p value |
| CKM stage  at 2011 | 1-0 | 1.73 | 0.309 |
|  | 2-0 | 2.05 | 0.043 |
|  | 3-0 | 2.08 | 0.018 |
|  | 4-0 | 3.91 | <0.001 |
|  | 2-1 | 1.18 | 0.080 |
|  | 3-1 | 1.20 | 0.094 |
|  | 4-1 | 2.25 | <0.001 |
|  | 3-2 | 0.98 | 0.999 |
|  | 4-2 | 1.87 | <0.001 |
|  | 4-3 | 1.90 | 0.004 |

| **ESM Table 6. The Association between CKM transitions and cognitive impairment with multiple comparisons** | | | |
| --- | --- | --- | --- |
|  | Pairwise comparisons | Estimates | Bonferroni-corrected p value |
| CKM transition between 2011 and 2015 | Improved vs. stable | 0.439 | 0.134 |
|  | Progressed vs. stable | 1.60 | 0.017 |
|  | Progressed vs. improved | 3.65 | 0.015 |

| **ESM Table 7. The Association between combined CKM transitions and cognitive impairment** | | | |
| --- | --- | --- | --- |
|  |  | Improved and stable | Progressed |
| Total population | Model 1 | 1.00 (ref) | 1.19 (1.02-1.81) |
|  | Model 2 | 1.00 (ref) | 1.23 (1.03-1.88) |
|  | Model 3 | 1.00 (ref) | 1.23 (1.03-1.89) |
|  | Model 4 | 1.00 (ref) | 1.75 (1.09-2.81) |
| Stage 0-1 at 2011 | Model 1 | 1.00 (ref) | 1.69 (0.72-3.93) |
|  | Model 2 | 1.00 (ref) | 1.70 (0.73-3.97) |
|  | Model 3 | 1.00 (ref) | 1.69 (0.72-3.93) |
| Stage 2 at 2011 | Model 1 | 1.00 (ref) | 2.52 (1.33-4.52) |
|  | Model 2 | 1.00 (ref) | 1.83 (1.11-3.39) |
|  | Model 3 | 1.00 (ref) | 1.87 (1.12-3.46) |
| Stage 3 at 2011 | Model 1 | 1.00 (ref) | 4.80 (1.82-11.83) |
|  | Model 2 | 1.00 (ref) | 5.18 (1.92-13.30) |
|  | Model 3 | 1.00 (ref) | 5.64 (2.05-14.87) |

Model 1: crude model

Model 2: adjusted for age and sex

Model 3: further adjusted for current drinking (yes/no), ideal physical activity (yes/no), married (yes/no), depression (yes/no) and quartiles of social determinants of health.
